# Addressing healthcare vulnerabilities in nursing homes: Insights from human rights monitoring in two Austrian provinces

**DOI:** 10.1007/s00508-024-02409-2

**Published:** 2024-08-09

**Authors:** Arkadiusz Komorowski, Theresa Rahel Demmer, Marianne Auer, Marianne Schulze, Gabriele Fischer

**Affiliations:** 1 Department of Psychiatry and Psychotherapy, Medical University of Vienna, Währingergürtel 18–20, 1090 Vienna, Austria; 2 Center for Public Health, Medical University of Vienna, Kinderspitalgasse 15, 1090 Vienna, Austria; 3 Faculty of Psychology, University of Vienna, Wächtergasse 1, 1010 Vienna, Austria; 4 International Human Rights Consultant, Vienna, Austria; 5 Commission 3, Austrian Ombudsman Board, Singerstraße 17, 1015 Vienna, Austria

**Keywords:** Nursing care, OPCAT, CRPD, Dementia, Long-term care

## Abstract

**Background:**

Current demographic changes bear challenges for national care systems due to higher life expectancy of older citizens. Largely cut off from society, nursing home residents are at risk for violence, neglect, and other potential human rights violations. This study aimed to investigate healthcare vulnerabilities in nursing homes and evaluate the Austrian National Preventive Mechanism (NPM).

**Methods:**

Between 2017 and 2019, 55 monitoring visits were conducted in 32 nursing homes. Study outcomes from two Austrian provinces included data on infrastructure, occupancy, staffing, resident’s demographics and medical conditions, as well as measures related to the functioning of the NPM.

**Results:**

Accessibility with mobility aids was sufficient in 87%, but assistance for persons with visual or hearing impairments solely in 20–40% of the institutions. An understaffing with nursing assistants (−5.2 full-time equivalents in Carinthia) and home helpers (−1.6 in Carinthia and Styria) was present. Less than 20% of the personnel received advanced training related to dementia and neuropsychiatric care. While 50% of the residents were diagnosed with a psychiatric disorder, approximately 36% received support from an appointed legal guardian. Of the monitoring visits 58.1% were conducted due to anonymous complaints and urgent referrals. The median processing times of the NPM and the provincial governments exceeded 250 days.

**Conclusion:**

Human rights monitoring reveals critical aspects in nursing home care, including insufficient accessibility, understaffing and inadequate training. Although the authorities’ handling times hinder prompt responses, the NPM may foster systemic improvements and accountability within nursing homes.

**Supplementary Information:**

The online version of this article (10.1007/s00508-024-02409-2) contains supplementary material, which is available to authorized users.

## Introduction

Individuals with severe psychiatric disorders, including affective and psychotic disorders as well as older citizens with dementia are frequently admitted to nursing homes due to a compromised physical and mental health [1, 2]. In light of recent demographic trends, implying an increased life expectancy and mounting global challenges, the number of persons requiring nursing home care is growing worldwide [3, 4]. While short-term stays in nursing homes focus on post-acute rehabilitation, long-term stays may also provide palliative and end of life care [5, 6]. The global population of individuals living with dementia already surpassed 50 million in 2020 [7]. In Austria the prevalence of dementia within the general population is expected to increase from 90,500 in 2000 to 290,000 by 2050. Current estimates range between 140,000 [8] and 145,000 persons [9], whereby around two thirds of the cases affect women. Globally, the scale of nursing home care varies widely depending on the economic structure, public healthcare services, family ties and other sociocultural aspects [10, 11]. While approximately 1.4% of the general population is being cared for in nursing homes in Sweden and the Netherlands, lower proportions were reported in Germany (1%) [12] and the United Kingdom (0.6%) [13]. According to Austria’s National Statistical Institute [14] a total of 96,458 persons (1.1%) received nursing home care in 2019, including 8256 persons in Carinthia and 17,487 in Styria. In all 9 Austrian provinces there were 870 nursing homes listed, whereby approximately half were publicly operated, one quarter consisted of private for-profit entities and the remaining facilities were operated by non-profit organizations (see Supplementary Table 1). Collectively, around 45,500 individuals were employed within the nursing home sector with a full-time equivalent (FTE) of 35,972, comprising approximately 13,100 registered nurses, 24,300 nursing assistants and 3500 home helpers [14, 15].

Nursing home residents are at risk of facing stigma and discrimination due to their dependence on caregivers [16, 17]. In order to ensure the provision of sufficient care, national health strategies must be guided by internationally accepted standards [18]. Alongside other UN documents, the *Convention against Torture and Other Cruel, Inhuman or Degrading Treatment or Punishment (CAT) *as well as the *Convention on the Rights of Persons with Disabilities (CRPD) *are central to the protection of persons in vulnerable circumstances. Both treaties impose essential obligations on States Parties to prevent, punish, and investigate incidents of abuse and ill treatment. To this effect, Austria established distinct commissions, governed by the Austrian Ombudsman Board (AOB), with the purpose of monitoring places where persons may be deprived of their liberty [19, 20]. Given the common seclusion of nursing homes, neglect and other forms of human rights violations appear especially relevant in these facilities [21]. Consequently, recent publications advise to address structural issues and interrelated factors in order to ameliorate the broadly conceived rights of nursing homes residents [17, 22].

Following the adoption and ratification of international treaties, independent human rights commissions are equipped with unique competencies to identify shortcomings within the healthcare system. However, there is a scarcity of research on this matter and the practical consequences of human rights treaties for individuals with psychiatric disorders and dementia remain uncertain. By evaluating healthcare vulnerabilities, this study aimed to investigate nursing homes in two Austrian provinces and further give insights into the functioning of the Austrian National Preventive Mechanism (NPM).

## Methods

### The Austrian National Preventive Mechanism (NPM)

The Austrian human rights catalogue, dating back to the Monarchy, is characterized by a sui generis culture of scattering qualified majority provisions throughout simple majority laws and supplemented by the *European Convention for the Protection of Human Rights and Fundamental Freedoms (ECHR)* that has constitutional status. The *Optional Protocol* to the *CAT (OP-CAT) *entered into force in June 2006, strengthening the protection of persons deprived of their liberty by nonjudicial means of a preventive nature at the national level. In acceding to the obligation to establish a NPM under *OP-CAT* in Austria, the responsibilities of the AOB, a Scandinavian-style Ombudsman Office established in 1977, were therefore broadened in 2012. The *CRPD*, which entered into force in May 2008, demands that States Parties ensure effective monitoring of facilities and programs designed to serve persons with disabilities (Article 16 (3)) and the establishment of national focal points to monitor the implementation of the Convention (Article 33 (2)). While international human rights treaties, save one anti-racism and a few child rights clauses, are not directly applicable, Article 148a (3) of the Austrian Federal Constitutional Act (*Bundes-Verfassungsgesetz B‑VG*) reflects the scope of the AOB’s mandate [23]. Stipulated by the regulations and rules governing the AOB, six independent commissions were given requisite independence and authority to conduct fact-finding in distinct regions (Commission 1: Tyrol and Vorarlberg, Commission 2: Upper Austria and Salzburg, Commission 3: Styria and Carinthia, Commission 4: Vienna, Commission 5: Lower Austria and Vienna, and Commission 6: Burgenland and Lower Austria). In 2021, a separate commission was established for nationwide monitoring of prisons, redefining prior responsibilities of the commissions regarding the penal system. The findings from the NPM are reported by the AOB annually to the national as well as federal councils and biannually to the provincial parliaments.

### Data collection and processing

Structured study data related to nursing home care were collected by Commission 3, and occasionally external experts, between 2017 and 2019. The original dataset was based on 55 standardized protocols recorded during human rights monitoring visits in Styrian and Carinthian long-term care facilities. Each protocol included personal observations as well as transcripts of interviews with residents, staff members and the management of the visited institutions. The number of individuals who were interviewed depended on the size of the facilities and the staffing level. All members of Commission 3 were trained in human rights monitoring and had multiple years of experience with the transcription of interviews. Only anonymized data without potentially identifying details from single individuals were analyzed.

### Study measures

The primary study measures reporting the findings from human rights monitoring in nursing homes were based on a protocol template defined by the AOB and the affiliated Human Rights Advisory Council in 2013 (see Supplementary Table 2). Each predefined protocol section comprised information related to the quality of care in terms of structure. Actual admissions and granted care places were compiled to calculate occupancy rates. Furthermore, accessibility for persons with disabilities, staffing, the qualification of the personnel (psychiatric education) and implementation of supervision were assessed. The population characteristics were assessed with respect to gender, forensic background and the proportion of legal guardians. Given the high prevalence of patients with psychiatric disorders admitted to nursing homes, diagnoses of all residents were screened for the presence of mental and behavioral disorders (F00–F99) according to the International Classification of Diseases (ICD), version 10 [24]. With respect to the monthly care allowances in Austria, ranging from € 162.50 (level 1) to € 1745.10 (level 7), residents were grouped into three advancing categories (< 3, 3–5 and >5). If available, additional information about living conditions was documented qualitatively.

In order to give insights into the functioning of the Austrian NPM, the frequencies, duration, periods and circumstances of the visits were assessed. After each visit, Commission 3 sent a protocol to the AOB that processed the protocol and subsequently sent a summarized legal assessment to the provincial government. Thus, the handling time of the commission was measured from the date of the visit until the recording of the protocol. The handling time of the AOB was measured from the date of submission of the protocol by Commission 3 until formal correspondence of the AOB with the responsible provincial government. The handling time of the provincial government was measured from the date of initial correspondence with the AOB until issuance of a concluding statement by the governmental authority.

### Statistical analysis

All data were analyzed retrospectively using descriptive statistics such as mean, standard deviation and variance. In cases of non-normally distributed data, the median (including minimum/maximum) was calculated. Quantitative outcomes were extracted from the protocols and coded using Microsoft Excel, Version 16.43 (Microsoft Corp., Redmond, WA, USA). Subsequent data analyses were performed using SPSS Statistics for Macintosh, Version 24.0 (IBM Corp., Armonk, NY, USA). In the case of multiple visits to an individual facility, only the initial visit was included in the analyses to avoid any bias. Where applicable, datasets were compared separately between Styria and Carinthia. Data that had not been collected during the monitoring visits were treated as missing values and excluded on a case-by-case basis. Missingness at random was assumed and available case analyses were performed (the number of included protocols is indicated by *N*).

The calculation of overstaffing and understaffing (compared to legal requirements) was based on full-time employment, whereby an FTE of 1.0 corresponded to the maximum number of working hours. The FTE was transformed from 38 h per week to 40 h per week, if necessary. Regarding the staffing ratio, legal regulations differed between both provinces: In Styria, required staffing ratios were calculated according to the care levels of the residents as defined by law. In Carinthia, the staffing ratio was not dependent on the care levels and therefore based on the total number of residents admitted to an institution.

## Results

### Healthcare vulnerabilities in nursing homes

According to data from the National Statistical Institute [14], Styria (population: 1.24 million; size: 16,399 km^2^) and Carinthia (561,293; 9537 km^2^) collectively accounted for approximately one third of Austria’s geographic size, representing a substantial part of the national population with dementia in 2019. Although both provinces differed with respect to the overall population and land area, the average age of the inhabitants as well as the life expectancy at birth were comparable (see Supplementary Table 3). In both provinces, a higher prevalence of nursing homes in rural areas compared to urban areas was observed.

Between 2017 and 2019 Commission 3 conducted 55 unannounced monitoring visits in 32 nursing homes, covering 24 (11.5%) of the existing facilities in Styria and 8 (10.1%) in Carinthia. The visited facilities, providing care for individuals dependent on caregivers, varied greatly in size (3–165 beds), with average occupancy rates of 97.8% (SD ± 11.7) in Styria and 91.4% (SD ± 16.1) in Carinthia (Table 1). Despite existing human rights norms in accordance with the *CRPD*, adequate accessibility for persons with disabilities was only partially present. Whereas accessibility with mobility aids such as wheelchairs was sufficient in 86.4% (19 facilities, *N* = 22 analyzed protocols; Styria) and 87.5% (7 facilities, *N* = 8 analyzed protocols; Carinthia) of the nursing homes, access for persons with visual or hearing impairments was established solely in 40.0% (4 facilities, *N* = 10 analyzed protocols; Styria) and 20% (1 facilities, *N* = 5 analyzed protocols; Carinthia).

**Table 1:** Healthcare vulnerabilities in Austrian nursing homes

	Styria	*N*	Carinthia	*N*
*Nursing home infrastructure*				
Number of beds (maximum capacities)	3–165	23	36–113	8
Occupancy rates (actual admissions; granted care places)	97.8% (± 11.7) (67.7 ± 45.7; 72.6 ± 52.1)	23	91.4% (± 16.1) (65.1 ± 21.8; 74.3 ± 26.4)	8
Sufficient accessibility for people with mobility impairments	86.4% (19 facilities)	22	87.5% (7 facilities)	8
Sufficient accessibility for people with visual or hearing impairments	40.0% (4 facilities)	10	20.0% (1 facility)	5
*Personnel*				
Average staffing with registered nurses	6.9 FTE ( ± 4.1) (+2.0 ± 2.9)	24	6.5 FTE (± 2.7) (+1.1 ± 1.5)	7
Average staffing with nursing assistants	16.7 FTE ( ± 12.1) (+2.9 ± 5.4)	24	13.9 FTE (± 8.9) (−5.2 ± 4.2)	7
Average staffing with home helpers	2.8 FTE (± 3.2) (−1.6 ± 2.0)	24	1.0 FTE (± 1.0) (−1.6 ± 1.5)	7
Proportion of registered nurses with advanced psychiatric education	13.1% (± 20.1)	17	20.0% (± 44.7)	5
*Demographics*				
Female residents	59.2% (± 18.9)	20	66.0% (± 11.6)	7
Forensic residents	10%	1	–	–
*Health care*				
Prevalence of psychiatric disorders	50.0% (± 34.1)	14	55.1% (± 40.8)	6

*N* indicates the number of evaluated protocols; available case analyses were performed; standard deviation is provided for metric variables; psychiatric disorders were classified according to the International Classification of Diseases [23]; average full-time equivalents (*FTE*) were compared to the legal requirements in both provinces (+ indicates overstaffing, – indicates understaffing)

**Fig. 1:**
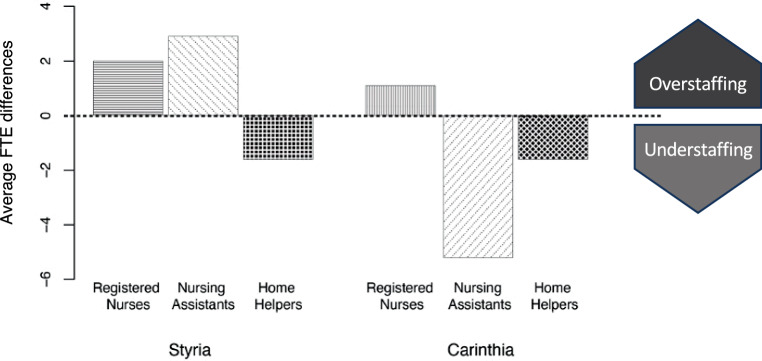
Average differences between actually existing and legally required full-time equivalents (FTE) of nursing home staff. While staffing of registered nurses and nursing assistants partly exceeded the standards stipulated by the provincial governments, shortages of home helpers were observed in both provinces

Addressing the challenging working conditions in nursing home care, shortages of home helpers were observed in both provinces (Fig. 1). In Styria, the FTE of registered nurses (6.9 ± 4.1) and nursing assistants (16.7 ± 12.1) exceeded the legal requirements set by the provincial government by 2.0 FTE (SD ± 2.9) and 2.9 FTE (SD ± 5.4), respectively. In contrast, an understaffing with home helpers (average staffing: 2.8 FTE, SD ± 3.2; deficit: 1.6 FTE, SD ± 2.0) was observed. In Carinthia, the FTE of registered nurses (6.5 ± 2.7) exceeded the legal requirements by 1.1 FTE (SD ± 1.5). However, an understaffing with nursing assistants (average staffing: 13.9 FTE, SD ± 8.9; deficit: 5.2 FTE, SD ± 4.2) and home helpers (average staffing: 1.0 FTE, SD ± 1.0; deficit: 1.6 FTE, SD ± 1.5) was present. Regarding possible strategies to facilitate the mental well-being of the personnel, only 17.4% (8 facilities, *N* = 46 analyzed protocols) of the nursing homes implemented a structured supervisory program, even though it provides a useful preventive measure for mediating personal conflicts or unexpected incidents. If available, the staff consistently attended periodical supervision meetings in just 6.5% (3 facilities, *N* = 46 analyzed protocols) of the monitored institutions. Regarding the training of the nursing home staff in Styria and Carinthia (*N* = 22 analyzed protocols), no more than 20% of all registered nurses had a specialized education with an emphasis on dementia and neuropsychiatric care.

While a higher rate of female residents was observed in Styria (59.2%, SD ± 19.0%) and Carinthia (66.0%, SD ± 11.6%), only one nursing home admitted persons formerly treated in forensic psychiatric health services (proportion of forensic residents: 10%). In both provinces, around half of the admitted residents were diagnosed with a psychiatric disorder and 35.7% (SD ± 20.9) received support from an appointed legal guardian to exercise their legal capacity. Furthermore, a substantial proportion of residents necessitated assistance concerning personal hygiene, hearing aids and mobilization. While care levels 3–5 (67.5%, SD ± 13.5) were observed most frequently, higher (> 5; 22.3%, SD ± 11.8) and lower (< 3; 10.5%, SD ± 12.8) care levels occurred less often.

Regarding the living conditions in nursing homes, the majority of residents criticized the daily schedule, in particular the early suppertime around 4:30 p. m. While supper was usually followed by night care provided by the staff before the end of their day duty, only very few facilities provided any form of entertainment in the evening hours. Although personal observations during the visits implied that undetected or untreated hearing impairments could result in reclusive behavior of affected residents, this study revealed no further presumptions related to abuse, neglect and degrading treatment.

### Independent human rights monitoring

Monitoring visits were conducted without prior announcement by at least two members of Commission 3 and, at times, additional external experts. The Commission consisted of a multidisciplinary team of one chairperson and seven members from various backgrounds, including law, medicine, nursing, psychology, education and others. In addition to monitoring nursing homes, further preventive activities of the NPM took place in various areas, such as child and youth welfare facilities, police detention centers, penal institutions and hospitals (see Supplementary Table 4).

In the majority of the cases the visits were conducted due to urgent referrals from other authorities or anonymous complaints (58.1%; 18 visits, *N* = 31 analyzed protocols). Such ad hoc visits were planned as soon as the NPM received information related to unsatisfactory nursing home care or insufficient capacities (e.g., a particular long waiting time until admission). Occasionally, monitoring visits to individual facilities were conducted multiple times over the years. While a total of 23 (41.8%) follow-up visits took place between 2017 and 2019, potential changes within the visited nursing homes were not evaluated in this study. Further information on the monitoring activity of Commission 3, including the time and duration of the visits, can be found in Table 2.

**Table 2:** Overview of the monitoring activity in nursing homes conducted by Commission 3

	Total number (%)	*N*
*Reason for monitoring visits*		
Urgent referrals	18 (58.1%)	31
Regular visits	13 (41.9%)	31
*Frequency of monitoring visits to individual facilities*		
Single visits	32 (58.2%)	55
Two visits	12 (21.8%)	55
Three visits	6 (10.9%)	55
> three visits	5 (9.1%)	55
*Duration of monitoring visits*		
Median (range)	4.2 h (1.0–6.6)	55
*Monitoring period*		
Daytime (before 6:00 p. m.)	37 (69.8%)	53
Evening (after 6:00 p. m.)	15 (28.3%)	53
Whole day (before and after 6:00 p. m.)	1 (1.9%)	53
Workday	41 (78.9%)	52
Saturday	5 (9.6%)	52
Sunday and official holidays	6 (11.5%)	52

*N* indicates the number of evaluated protocols; available case analyses were performed; all facilities were visited between 2017 and 2019

**Fig. 2:**
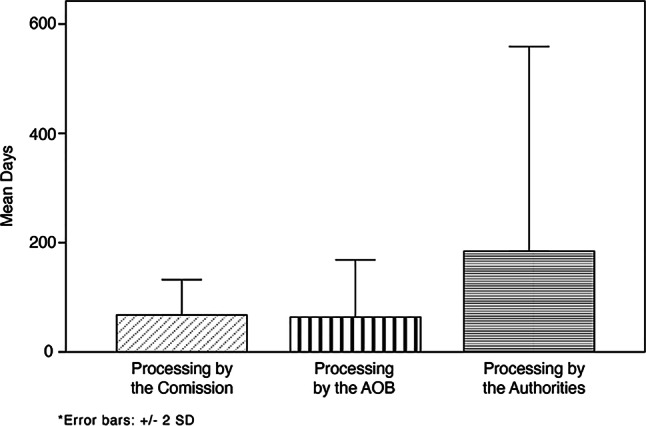
Handling times of Commission 3, the Austrian Ombudsman Board (AOB) and the provincial governments. While the handling time of the commission was comparable to the one of the AOB, the handling time of the provincial governments was longer. Remarkably, two individual protocols were processed by the Styrian authorities for more than 800 days

Noteworthy, the total processing time for the Austrian NPM, reflecting the typical duration from the day of the visit to the official reply by the provincial governments, combined to an average of 312.4 days (SD ± 199.5; median: 255 days, range: 115–1072) (Fig. 2). This suggests room for improvement in the efficiency and functioning of the NPM in handling the respective cases. The time periods referring to the recording of the protocols by Commission 3 (mean handling time: 67.5 days, SD ± 32.3; median: 65 days, 6–161) and processing of the protocols by the AOB (mean: 63.9 days, SD ± 52.2; median: 57 days, 7–235) were comparably short. In contrast, the handling times of the provincial governments were remarkably long (mean: 187.3 days, SD ± 187.3; median: 120 days, 41–1001), with exceptionally lengthy processing periods for multiple outliers (more than 2 years).

## Discussion

Based on human rights monitoring, this study investigated healthcare vulnerabilities in Styrian and Carinthian nursing homes over 3 consecutive years. Apart from partially inadequate accessibility for persons with disabilities, especially those with visual or hearing impairments, understaffing and insufficient training related to dementia and neuropsychiatric care were present in both provinces. This underscores the importance of addressing the needs of nursing home residents and the necessity for adequate healthcare in such settings. While no acute cases of abuse, neglect or degrading treatment were observed during the study period, the relatively long processing time of the NPM limited short-term actions related to nursing home care in Austria.

### Critical aspects in nursing home care

The rising rates of dementia and other psychiatric disorders are placing additional strain on already pressured care systems in Austria [11, 25], especially concerning the recruitment and retention of specialist personnel such as nursing, psychological and medical staff. In addition to maintaining good physical and mental health, the quality of life is influenced by active participation and involvement as highlighted by the World Health Organization [26]. The *CRPD*, with a specific focus on Article 19 (*living independently and being included in the community*), further advocates the protection and promotion of the right to social participation [27]; however, the limited availability of public transport in rural areas in Styria and Carinthia can pose obstacles to the mobility of nursing home residents and thus limit access to support networks in the immediate vicinity of long-term care facilities.

In 2019 the National Statistical Institute reported that 8229 persons were employed within the nursing home sector (6307 FTE; 85.9% female) in Styria and 3022 employees (2420 FTE; 88.5% female) were registered in Carinthia [14]. The understaffing found in this study is in line with commonly reported staff shortages in Austrian nursing homes which also limit occupancy rates [28, 29]. Given that the coronavirus disease 2019 (COVID-19) pandemic further aggravated staff shortages in the nursing home sector [30], even lower occupancy rates and longer waiting times can be assumed in the future. Regarding appropriate qualifications of the personnel in Styria and Carinthia, this study showed that no more than a few care providers employed staff specializing in dementia care. Given the complexity of psychiatric nursing, the relatively low proportion of registered nurses with advanced training focusing on neuropsychiatric disorders appears to be insufficient. This shortcoming seems particularly relevant for residents who were previously admitted to forensic psychiatric health services, as they require specialized care and support [31]. Moreover, Article 4 (i) of the *CRPD* demands the promotion of training for professionals working with persons with disabilities to ensure assistance in line with the Convention [32] and the AOB advises the engagement of nurses with specialized psychiatric training to provide better care for nursing home residents with mental health conditions [33].

Usually, more than 70% of nursing home residents are diagnosed with dementia [6, 13]. Compared to other countries, previous studies showed a slightly lower prevalence of dementia diagnoses (approximately two thirds) but higher rates of psychiatric comorbidities in Austria [34, 35]. Accordingly, this study revealed that approximately 50% of the residents were diagnosed with a neuropsychiatric disorder and approximately one third received support from an appointed legal guardian due to an impaired capacity to declare one’s will. To meet the standards of the *CRPD* and to reduce the overall high rate of legal guardians in Austria [36], the previous *Guardianship Act* was significantly rewritten in a participatory process in 2018, including courts, lawyers, the AOB and, most notably, persons with disabilities as experts in their own right. Still, besides impaired self-determination, nursing home residents were subjected to an extra early bedtime and a lack of entertainment in the evening hours, provoking the feeling of loneliness. Social isolation, however, increases the risk for cognitive decline, especially in persons with untreated hearing impairments [37, 38].

### Strengths and weaknesses of the Austrian NPM

Regarding nursing home care, the Austrian NPM established oversight mechanisms to protect the rights and well-being of individuals in vulnerable circumstances [20]. According to the rules of procedure of the AOB, the commissions are obliged to submit a protocol within 4 weeks after the day of the visit; however, the actual handling time of Commission 3 markedly exceeded this time allowance. Although unrestricted access to documents had to be granted during the monitoring visits, subsequent requests for information, including demographic data and staffing levels, usually delayed the completion of the protocols. Due to the necessity to provide legal assessments of the submitted protocols, considering both previous and current visits, the potential to reduce the handling time of the AOB appears limited. The main bottleneck is the prolonged processing time of the provincial governments, undermining the monitoring mandate of the NPM. Given that considerably long handling times compromise immediate actions and preventive aspirations, a comprehensive human rights monitoring framework considering cultural, financial, legal and political factors may prove to be more advantageous [22, 27].

### Limitations of the study

Donabedian’s concept of evaluating quality indicators on three levels, structure, process and outcome, serves as a comprehensive approach to measure the quality of care [39]. The data used in this study, which relied on a protocol template of the NPM, held considerable value but also limited the analysis of measures associated with the process and outcome levels of nursing care. As more than half of the visits were conducted due to urgent referrals based on first-hand information from relatives and former employees, a negative bias regarding care conditions cannot be ruled out. The main limitations related to the fact that monitoring visits were not uniformly conducted throughout Austria due to the autonomy of each commission. The other commissions predominantly documented qualitative data on the well-being of residents and staff, largely leaving out structural information. Thus, the present findings are mainly representative for Styria and Carinthia and can only to a certain degree be generalized to other regions. The apparent gap between the number of visited facilities compared to the total number of existing nursing homes in Styria and Carinthia can be ascribed to the restricted (time) resources of Commission 3. Given that all members work on a part-time basis, monitoring all 288 nursing homes in both provinces will require several years more. While the majority of the nursing homes were monitored according to the superordinate monitoring strategy of the Austrian NPM, multiple protocols were incomplete and missing data hampered statistical evaluation.

## Conclusion

Progressively decreasing family care resources combined with overburdened care systems pose significant challenges for society in Austria and other countries. Integrating individuals with psychiatric disorders and dementia into the community through appropriate social support constitutes an important aspect for improving the well-being of those in vulnerable circumstances, as mandated by the *CRPD* as well as the *CAT* and its *Optional Protocol*. Insufficient accessibility, understaffing and inadequately educated employees can lead to ill-treatment, negligence or violence towards nursing home residents. This study underscores the crucial role of the NPM in revealing the critical conditions and constraints prevalent in nursing home care. Importantly, adequate efforts and financial resources are essential to maintain preventative human rights monitoring and facilitate timely interventions in the future.

## Supplementary Information

Supplementary Tables 1, 2, 3 and 4

## Data Availability

The data that support the findings of this study are available on request from the corresponding author with the permission of the Austrian Ombudsman Board. The data are not publicly available due to privacy restrictions.
