# Supplementary material for: Addressing healthcare vulnerabilities in nursing homes: Insights from human rights monitoring in two Austrian provinces
Source: Wien Klin Wochenschr. 2024 Aug 9;137(11-12):368–76. doi: 10.1007/s00508-024-02409-2 (PMC12176916; doi:10.1007/s00508-024-02409-2)
Supplement: Supplementary file 1 — Supplementary Tables 1, 2, 3 and 4 [file 508_2024_2409_MOESM1_ESM.docx]

**Supplementary material:**

Supplementary Table 1: Privately-run and public nursing homes in Austria.

|  | **Privately-run** | **Public** | **Total** | **Source** |
| --- | --- | --- | --- | --- |
| Burgenland | 38 (88 %) | 5 (12 %) | 43 | Provincial Government of Burgenland, 2021 |
| Styria | 178 (85 %) | 31 (15 %) | 209 | Styrian Provincial Government, 2020 |
| Carinthia | 61 (77 %) | 18 (23 %) | 79 | Carinthian Provincial Government, 2020 |
| Vienna | 51 (56 %) | 40 (44 %) | 91 | BMASGK, 2018; Court of Audit Austria, 2020 |
| Lower Austria | 48 (48 %) | 53 (52 %) | 101 | Lower Austrian Provincial Government, 2019 |
| Vorarlberg | 23 (45 %) | 28 (55 %) | 51 | Provincial Government of Vorarlberg, 2019; Court of Audit Austria, 2020 |
| Upper Austria | 28 (21 %) | 108 (79 %) | 136 | Upper Austrian Provincial Government, 2019 |
| Salzburg | 11 (15 %) | 63 (85 %) | 74 | Court of Audit Austria, 2020 |
| Tyrol | - | - | 98 | Court of Audit Austria, 2020 |

*Footnote: In Tyrol, only the total number of nursing homes was reported.*

Supplementary Table 2: Protocol template defined by the Austrian Ombudsman Board (AOB) and the affiliated Human Rights Advisory Council.

| **Predefined protocol sections** | **Information related to the quality of care** |
| --- | --- |
| Location of the facility | Accessibility by public transportation |
| Structure of the building | Accessibility for persons with disabilities |
| Equipment of the facility | Sanitary installations; interior decoration; reachability of staff |
| Living condition | Hygiene standard; boarding; cultural and recreational activities; public life; religious freedom |
| External relations | Contact with (legal) representatives; visitations; ban on visitors |
| Family and private sphere | Right to privacy |
| Employment and education | Occupational and educational opportunities |
| Internal relations | Language; access to information and interpretation; rights and obligations; house regulations |
| Complaint management | Handling of complaint |
| Restriction of freedom | Isolation; (medical) restraint; documentation; principle of proportionality |
| Safeguards | Presence or use of weapons; security service |
| Torture and other cruel, inhuman or degrading treatment | Accusations and reactions; preventive measures |
| Health care | Health condition; staffing |
| Personnel | Qualification; education; supervision; working conditions |

Supplementary Table 3: Demographic characteristics in Styria and Carinthia.

|  | Styria | Carinthia |
| --- | --- | --- |
| Overall population 2019 (year average) | 1,244,474 | 561,062 |
| Area (square kilometers) | 16,399 km² | 9,536 km² |
| Population density (inhabitants per square kilometer) | 75.9 | 58.9 |
| % of persons over 65 years in 2019 | 20.5 % | 22.1 % |
| Average age of the population | 44.1 | 45.2 |
| Life expectancy at birth according to gender in 2019 | Women: 84.7  Men: 79.5 | Women: 84.7  Men: 79.5 |
| Persons receiving in-patient care in 2019 | 17,487 | 8,256 |
| % of persons receiving in-patient care | 1.4 % | 1.5 % |
| Total number of nursing facilities in 2019 | 209 | 79 |

*Footnote: Demographic data were based on information provided by Austria’s National Statistical Institute [16].*

Supplementary Table 4: Human rights monitoring within the scope of the Austrian National Preventive Mechanism (NPM) (from 22.08.2012 until 31.12.2020).

|  | **Commission 1** | **Commission 2** | **Commission 3** | **Commission 4** | **Commission 5** | **Commission 6** | **Total** |
| --- | --- | --- | --- | --- | --- | --- | --- |
| Regional responsibility | Tyrol & Vorarlberg | Upper Austria & Salzburg | Styria & Carinthia | Vienna | Lower Austria & Vienna | Burgenland & Lower Austria |  |
| Nursing homes | 163 | 133 | 167 | 109 | 144 | 189 | **905** |
| Child and youth welfare facilities | 94 | 69 | 77 | 146 | 188 | 135 | **709** |
| Police detention centers and Police stations | 81 | 157 | 112 | 124 | 67 | 58 | **599** |
|  |  |  |  |  |  |  |  |
| Correctional institutions | 42 | 47 | 69 | 74 | 54 | 46 | **332** |
| Coercive acts by the executive authorities | 48 | 62 | 50 | 82 | 47 | 15 | **304** |
| Hospitals | 7 | 1 | 37 | 10 | 31 | 6 | **92** |
| Psychiatric hospitals | 64 | 11 | 64 | 46 | 14 | 20 | **219** |
| Round-table discussions | 13 | 1 | 23 | 5 | 10 | 10 | **62** |
| All activities | 512 | 481 | 599 | 596 | 555 | 479 | **3222** |
